# Social prescribing for individuals with mental health problems: An ethnographic study exploring the mechanisms of action through which community groups support psychosocial well-being

**DOI:** 10.12688/wellcomeopenres.20981.1

**Published:** 2024-03-19

**Authors:** Henry Aughterson, Daisy Fancourt, Helen Chatterjee, Alexandra Burton

**Affiliations:** 1 Behavioral Science and Health, University College London, London, England, UK

**Keywords:** social prescribing, mental health, community, severe mental illness, mechanisms, complexity, ethnography

## Abstract

**Background:**

Social prescribing involves connecting individuals to community groups and activities, often to support their mental health and well-being. It has received increasing support in recent years across the NHS. There is a strong evidence base for the benefits of different types of community activities, including exercise groups, arts groups and nature interventions, on mental health outcomes, however, less is known about *how* these groups impact mental health and well-being. This study explores through what individual-level *mechanisms* (the ‘how’) these groups support psychosocial well-being.

**Methods:**

An ethnographic study was conducted over 12-months to explore key shared, individual-level mechanisms across 4 social prescribing community groups: football, singing, gardening and reading. This study focused mostly on those with severe mental illness, whereas previously most social prescribing studies have focused on mild to moderate mental health problems. To frame the findings, a ‘multi-level theoretical framework of mechanisms of action’ of leisure activities was used.

**Results:**

Key shared psychological mechanisms were: increased self-confidence and self-esteem, increased purpose/meaning, increased sense of achievement, experience of pleasure; social mechanisms included: increased social support, formation of friendships and reduced loneliness, enhanced sense of community and belonging; behavioural mechanisms were: increased independence and openness to experience, reduction in addictive behaviours and building healthier habits, increased work-seeking behaviour, and provision of structure & routine.

**Conclusions:**

It is hoped that the findings of this study can help referring professionals increase their understanding of exactly how such groups support individuals’ mental health, thus enhancing referring practices.

## Plain Language Summary

This study uses ethnographic methods, wherein the lead researcher spent over a year participating in 4 different community groups, using interviews, conversations and observation to explore the mental health impact of such groups on individuals' lives. The participants had moderate to severe mental health conditions, and the groups consisted of a reading, gardening, singing and football group. The study explored 'mechanisms' underlying the mental health benefits of these groups. Key shared psychological mechanisms were: increased self-confidence and self-esteem, increased purpose/meaning, increased sense of achievement, experience of pleasure; social mechanisms included: increased social support, formation of friendships and reduced loneliness, enhanced sense of community and belonging; behavioural mechanisms were: increased independence and openness to experience, reduction in addictive behaviours and building healthier habits, increased work-seeking behaviour, and provision of structure & routine. It is hoped that the findings of this study can help referring professionals (e.g. GPs, social workers, link workers) increase their understanding of exactly how such groups support individuals’ mental health, thus improving referring skills.

## Introduction

Social prescribing is one approach to addressing rising mental health problems and loneliness. It involves the referral of individuals to community groups and activities, such as community gardening, choirs, reading clubs, walking groups, and volunteering ( Drinkwater *et al.*, 2019). The General Practitioner (GP)-link worker social prescribing referral model (involving GP-referral of a patient to a ‘link worker’ who then co-produces a social prescription with the patient) has received significant attention and funding, and early evidence suggests it can support mental health, alleviate GP workload and create more time for a personalised approach with patients ( Moffatt *et al.*, 2017; Woodhead *et al.*, 2017). Indeed, National Health Service (NHS) England has funded 4500 link workers across the United Kingdom (UK) ( NHS England, 2019). However, there are many possible referral mechanisms into community groups, including social care, mental health charities, leaflets/posters, or word-of-mouth (self-referral).

There is growing evidence that engagement in community groups can support psychosocial well-being and mental health among those with mild to moderate mental health problems ( Abbing *et al.*, 2018; Ascenso *et al.*, 2018; Clatworthy *et al.*, 2013; Coulton *et al.*, 2015; Daykin *et al.*, 2018; Fancourt & Tymoszuk, 2019; Jones *et al.*, 2013; Long & Stavel, 1995; Martin *et al.*, 2018; Petruzzello *et al.*, 1991), with fewer studies focusing on individuals with severe mental illness, but some evidence suggesting benefits e.g. improvements in anxiety symptoms and quality of life ( Chang *et al.*, 2018; Saavedra *et al.*, 2018). Most of these studies have explored the impact on stress, anxiety, and depression *outcomes*. However, the Medical Research Council (MRC)’s guidance on complex interventions has called for a greater understanding into the *mechanisms* (or causal processes) underlying the outcomes or benefits of such interventions, in order to advance research ( Moore *et al.*, 2015). (e.g. if an *outcome* is “reduced depression score”, then one *mechanism* helping to lead to such an outcome might be “development of a healthy habit”).

Some studies have identified isolated mechanisms underlying the impact of social prescribing activities on mental health, including mechanisms such as increased social connectedness, heightened self-esteem, increased patient activation, greater optimism, and improvement in health-related behaviours across a range of groups including music, exercise and arts groups ( Chang *et al.*, 2018; Clark *et al.*, 1998; Kellezi *et al.*, 2019), but rarely do studies attempt to capture the full mechanistic complexity of these activities – which often likely involve many different interacting mechanisms. Given that social prescribing groups are complex systems, it is likely that multiple mechanisms are involved, interact, and are additive to one another ( Craig *et al.*, 2008). The mental health *outcomes* of such activities (e.g. reduced anxiety) likely arise through the interactions of these mechanisms, which are activated by multiple *ingredients* (specific components within an activity), mediated by various *moderators* (individual-level and wider environmental, economic, and cultural factors that can affect engagement, as well as which mechanisms are activated for whom) ( Fancourt *et al.*, 2021).

We have found no studies that have used a broad theoretical framework to explain this complexity; though there have been studies using theoretical frameworks that take a more targeted focus e.g. the “social cure” or “social identity” frameworks ( Kellezi *et al.*, 2019; Wakefield *et al.*, 2020). In order to help frame and categorise a large number of mechanisms, we used the ‘multi-level leisure mechanisms framework’, which proposes that leisure (or social prescribing) activities can activate a large number of processes organised as biological, psychological, social and/or behavioural, which can then lead to improvements in health and wellbeing ( Fancourt *et al.*, 2021). These mechanisms can be studied flexibly at the individual (micro), group (meso) and/or societal (macro) level - our focus in this study was on the individual (micro) level, which we felt ethnography was particularly well suited to, allowing us to focus on the lives of the individuals across the groups. The framework was developed from a literature review conducted across multiple disciplines including psychology, neuroscience, medical sociology, anthropology, social epidemiology, and behavioural science which identified over 600 potential mechanisms of action for health within leisure activities. The use of this framework can help us to understand and capture the complexity underlying social prescribing activities.

A previous ethnography on "social prescribing" has been conducted ( Gibson *et al.*, 2021) with a deliberately specific focus on classed inequality, but, to our knowledge, no ethnographic study has explored social prescribing *mechanisms* (underlying the mental health impact on individuals). This methodology is particularly well-suited to studying individual level mechanisms as it enables the researcher to get closer to the lived experience of those attending social prescribing groups than other methodologies can offer, over an extensive period. It enables closer and more trusting relationships to form between researcher and participants, leading to the possibility of in-depth, rich findings.

There are many reasons why it is important to examine mechanisms and thus explore, in greater granularity, precisely *how* engaging in community groups impacts psychosocial well-being; it may be helpful for professionals involved in referring patients into such groups and who might not have first-hand experience of how they work ( Aughterson *et al.*, 2020). By enhancing the understanding of *how* these groups support people, this can also be better explained to potential beneficiaries. It might also help professionals to match patients/clients to groups, for example if these groups help reduce addictive behaviours, then someone with a history of substance misuse might be more readily considered. Moreover, the findings can support community groups to identify and explain the potential mechanisms underlying the benefits of their activities to funders and referrers, as well as consider new mechanisms that might be possible within their activities but are not currently being utilised fully, helping to design more targeted community-based interventions. It can also help inform evaluations of social prescribing interventions to measure the full impact of an activity and advance research.

Therefore, this study explored mechanisms of action across four different social prescribing community groups for adults with mental health problems. Mechanisms were explored fully within each group however this study focuses on the *shared, individual-level* mechanisms between the groups. This is the first known study to examine mechanisms of action across multiple social prescribing groups using an ethnographic approach.

## Methods

### Study design

This study uses qualitative, ethnographic methods. Ethnography literally means ‘writing about people’ (from the Greek words ethnos (people) and grapho (writing)) and using participant observation and other methods such as field notes and interviewing to explore, ‘on-the-ground’, the sociocultural lives of groups of individuals. The unique ability of ethnography to get closer to the lived experience of participants allows for the possibility of richer, more grounded data ( Davies, 2007). This does not deny the inevitability of ‘observer effects’ and ‘researcher bias’, and indeed a significant component of ethnography involves the reflexivity of the researcher; a ‘turning back on oneself’ in order to consider one’s positionality ( Salzman, 2002). The extended length of time the researcher spends with a social group allows the use of longer and more frequent interviews, conversations, and observations of participants with whom the researcher has formed relationships and gained a significant degree of trust, setting it apart from most qualitative interview or quantitative studies that have predominated this topic so far.

The ethnography adopted an open, inductive approach to explore the multiple effects of community groups on participants' lives through detailed observation, interpretation, and interviews. Inductive approaches work “from the bottom-up, using the participants’ views to build broader themes”, rather than testing a pre-defined theory (deductive) ( Soiferman, 2010). To avoid a narrow or overly prescribed focus, this study was primarily centred around the community group or activity itself, capturing participants from varied referral routes. Moreover, whilst previous studies have tended to focus on ‘mental health outcomes’, this study aimed to explore the wider social, psychological, and behavioural lives of participants, in terms that are meaningful to them, rather than through a strictly professional or medicalised lens. It is important to do this, otherwise the scientific community risks misunderstanding the complex and varied impact these groups can have on people’s lives, when merely focusing on the degree to which they reduce anxiety or depression symptoms. Building on this, and in keeping with a salutogenic approach ( Lindström & Eriksson, 2005), we were less focused on what the mental illness was (i.e. schizophrenia vs BPD) and more on the severe nature of illness or suffering that the individuals were experiencing (many of whom experienced multiple overlapping mental health conditions) and the positive mechanisms that were most important across these range of experiences. We do acknowledge a comparison (by type of mental illness) may add value; however, it might be more suited to certain quantitative methodologies, studies with a greater number of participants, or those which set out specifically to explore those differences by mental illness from the outset.

### Setting and participants

All four community groups were based in London, England. The groups were: 1) a football group for those with substance misuse and/or mental health problems, 2) a mental health choir, 3) a reading group and 4) a gardening group. The latter two groups, whilst less ‘mental health-related’ in their descriptions, were chosen because they still largely support members with a wide range of mental health difficulties. The groups were deliberately different to one another in order to better capture of the varied nature of social prescribing activities. They were purposively chosen to correspond to four of the main categories within which most social prescribing community groups broadly fall (excluding financial/welfare support): sports/exercise, nature, education, and the arts. Given the diversity of providers across the community’s ecosystem, no wider conclusions can be definitively drawn about the nature of *all* community groups/activities from the analysis of these four groups, but the purposive choice here does allow some useful comparisons and discussions into what some of the shared properties or differences between groups can be.

The research took place at a football centre, a community garden, a rehearsal room within a mental health hospital, a church, and (for two of the groups for several months due to Covid-19 restrictions) over video call (Zoom and Facebook Messenger Rooms). Interview participants were purposively sampled from the groups to obtain a good spread across gender, ethnicity, age, and mental health condition. All group members included in the study had a diagnosed mental health condition and/or significant psychosocial challenges and were able to give informed consent and communicate in English. Three active group referrers (two social prescribing link workers and a mental health nurse/fitness instructor), and four community group staff members (two group leaders/facilitators and two group directors/founders were also interviewed. Other participants were observed and conversed with over the course of the research but were not formally interviewed.

All participants were given a Participant Information Sheet and encouraged to ask questions in-person or over phone or video call, and written informed consent was then obtained. This was not possible with all members of the groups e.g. new members joining throughout the study period, or those who did not wish to be actively involved in the study, but these members were made aware of the researcher’s role and were not included in any field notes or interviews. All study participants were given pseudonyms and fully anonymised within the text.

### Data collection

The ethnography was conducted between December 2020 and December 2021, with HA (lead researcher) attending at least one session of each group per week, equating to an average of 3 hours per group every week and over 600 hours of observational data across the fieldwork period (see Table 1).

**Table 1.** Observation details and interview participants.

Activity	Average observation time (hours per week)	Total observation time (hours)	Interview participants
Football	3.5	175	7 club members; 1 referrer and member, 1 CEO/founder
Gardening	3	150	4 volunteers/members, 2 referrers (SP link workers), 1 CEO/director
Reading	3.5	175	6 group members, 2 facilitators
Singing	2.5	125	7 group members, 1 choir leader

Immediately after each group session, HA wrote field notes based on observations, participation and conversations with members and staff related the participants’ psychosocial health and their group engagement, using a note-taking software called Evernote, which allows efficient organisation of extensive notes via phone or laptop with automatic synchronisation.

During each observation HA adopted the role of ‘active participant’ ( Johnson *et al.*, 2006) – that is, whilst known by all group members as a researcher, they also actively took part fully in the activities of each group (e.g. playing football, singing, gardening and group reading). A wide range of participants were interacted with, but over time, as is often the case with ethnography, a sample of long-term research companions with whom a closer relationship had developed were focused on and selected for more formal interviews.

28 interviews were conducted, after 3 months of active participation, with 21 group members (see Table 2 for participant characteristics), three referrers and four staff members. The average length of interview was 72 minutes (range 43 to 108). These were semi-structured, open interviews, conducted mostly in-person before or after group sessions, however when more convenient for participants or due to Covid-19 restrictions, telephone or video calls were deployed. Interview topic guides were developed by all study authors, who span the fields of Behavioural Science and Health, Biosciences, and Arts and Health to explore the impact of the groups on participants' social lives, health-related behaviours, mental health, and wellbeing (see *Extended Data* ( Aughterson, 2024)). This included exploring how mental health problems had impacted participants’ lives until this point, their experience of mental health services, how and why they joined the group, any barriers to joining, why they keep coming back, what psychological and behavioural changes they have noticed in themselves since attending, etc. The interview format, however, was open, and participants were encouraged to talk at length about the group’s impact on them, in ways that they found most meaningful, rather than solely based around the structure of a pre-defined topic guide.

**Table 2.** Participant characteristics of the 21 group members.

Age	18–29	2
	30–39	2
	40–49	6
	50–59	6
	60–69	5
	70–79	2
Gender	Male	10
	Female	11
Ethnicity	White	12
	Ethnic minority	9
Employment	Unemployed	17
	Part-time	3
	Full-time	1
Mental health condition *	Depression	11
	Anxiety	6
	Post-traumatic stress disorder (PTSD)	4
	Schizophrenia	4
	Bipolar disorder	4
	Schizoaffective disorder	1
	Post-natal depression	1

*some participants reported multiple mental health conditions

### Data analysis

Data were collected through a combination of formal interviews, informal conversations and participant observations. Interviews were audio-recorded and transcribed using an encrypted AI software, Scrintal. The interview transcripts and field notes were imported into NVIVO 12, a qualitative data analysis software, along with text from emails, social media, and community group websites, as is common with ethnography which often combines multiple data sources. The data were analysed using reflexive thematic analysis, due to the flexibility it allows in analysing and interpreting patterns and shared meaning across diverse large data sets, as well as its capacity to incorporate multiple data sources ( Braun & Clarke, 2021a; Braun & Clarke, 2021b). The steps set out by Braun and Clarke were followed including firstly reading through the data sources and becoming familiar with the data, generating and defining codes, theme identification and producing the report ( Braun & Clarke, 2006). To generate codes, an inductive coding approach ( Thomas, 2006) was used, whereby HA developed and assigned codes to segments of text within the observation notes and interview transcripts that pertained to the research questions and which captured the essence of descriptions and participant experiences, often using words taken directly from participant accounts. HA then grouped codes into similar concepts and assigned overarching mechanistic theme names to each group of codes, with each theme reflecting a meaningful and distinct pattern in the data. Final themes were discussed within meetings and agreed upon by all study authors. From this approach, key mechanisms were identified within each group, and were then cross-matched across the four groups to identify key shared mechanisms. The final themes decided upon were mechanisms that were deemed ‘most important’ throughout the ethnographic fieldwork and interviews, specifically those that were observed or mentioned by participants most frequently or prominently AND appeared across participant accounts from three or four community groups.

Participant quotations and extracts from field notes are used throughout the *Findings* section to support the themes. The data were coded inductively, but in the analysis and write-up stages, the ‘multi-level theoretical framework of mechanisms of action’ was used, which is a framework built on the most extensive review of mechanisms involved in leisure activities to date ( Fancourt *et al.*, 2021) to categorise mechanisms into Psychological, Social, and Behavioural. The ‘Biological’ has been excluded from this study because most mechanisms within this category (e.g. altered immune function, modulating cardiovascular factors, and epigenetic changes) are not possible to explore adequately through qualitative, ethnographic means. Our study was focused on the micro-level (not meso- or macro-) mechanisms, which was the focus throughout the ethnographic observations and interviews.

### Ethics

Following transcription, original recordings of interviews were deleted, all participants and names of people mentioned in the transcripts were given pseudonyms to preserve their anonymity, and all specific locations and identifying features removed. The study was reviewed and approved by the Camden & Kings Cross Research Ethics Committee (279076; 20/LO/1214; 14.12.2020). Capacity to consent to be involved in the study applied the principles of the four-step Mental Capacity Act ( SCIE, 2017).

## Results

**Figure 1.**
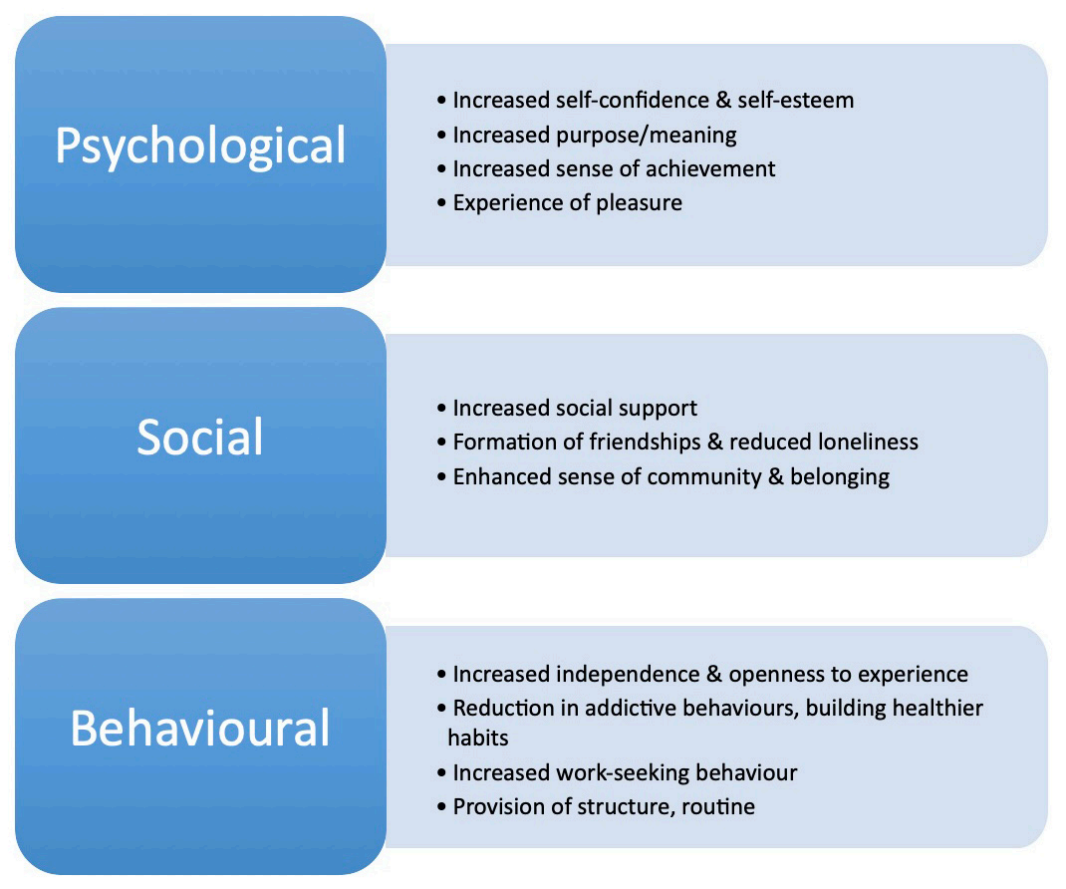
Table of shared mechanisms across the groups.

Mechanisms are organised and presented below using the multi-level leisure mechanisms framework headings: Psychological, Social, and Behavioural (See Figure 1). Participant quotes and field notes are used to help ground the themes in the data. Where quotes from individuals were obtained during interviews, they are labelled ‘INT’, and from informal conversations ‘INF’. Detailed explanations and features of each of the community groups (football, choir, reading and gardening) can be found as *Extended Data* ( Aughterson, 2024).

## Mechanisms

### Psychological

***Increased self-confidence & self-esteem.*** Group members experienced improvements in self-esteem and self-confidence, because of their engagement in community activities. Before joining the gardening group, Anna, who has ongoing PTSD, experienced eighteen months of dramatically reduced self-confidence, self-esteem, severe social anxiety, and depression. She spent this time at home by herself, not talking to or seeing anyone, fearful of socialising and leaving the house. She felt the gardens were the main factor in slowly building up her self-esteem and confidence, which in turn drastically improved her levels of social anxiety and depression. It acted as a “ *stepping-stone”* for engaging with the wider world, and moving on with her life:

*My confidence and self-esteem were the biggest stumbling blocks for me through everything I wanted, like thinking would I even be able to work again… the whole gardens group has meant my self-esteem is much, much better.*
*It was a secure, solid stepping-stone. I’ve stepped on the stone and I’ve looked around and thought, oh my foot is in the water, Oh it only comes up to my ankles and it*’ *s safe. There are no nasty things in there. That*’ *s really the analogy*.
*It*’ *s interesting how it took getting me out into the gardens, fresh air, a welcome weekly commitment to look forward to, for me to make such headway in getting involved in the world at large again.*
(Email extract sent from Anna to staff at the Gardens)

Sammy, who had been sectioned in the local mental health hospital for 6 months following a diagnosis of paranoid schizophrenia, and whose weight increased by 50kg during his hospital stay which he attributed to medication side effects and not being able to exercise, also experienced significant confidence improvements, from attending the football group:

*It*’ *s really helped my confidence. Because of my weight gain I was very shy, I didn’t want to go out and was worried about what other people thought and would think about me. But I found that being around other people (here), it*’ *s completely the opposite, that people don*’ *t care what way you are, as long as you’ve got a good heart and you’ve got a passion for the game, it kind of means they accept you rather than just whipping you or saying that you shouldn’t be playing or stuff like that.* (Sammy; INT)

***Increased sense of achievement.*** The groups provided many members with a regular sense of achievement, for example with the choir this was often related to the hard work and training that goes into learning songs and performing them in front of a crowd in a venue, as Wendy (who joined the choir to help with bereavement) explains:

*I suppose it's like training, isn't it? You've trained for something, and there's a goal in mind, and it's been good the training, it's been hard work, but at the end of the day, if you've achieved what you set out to do, it's that little bit about 'well done'. I think that's why they like performing, because it's like we've got something to aim for. And then we've achieved it and there's a feeling of satisfaction. Like you've won a gold medal, you know, it feels like that.* (Wendy; INT)

Wendy also made the important point, that for members with severe mental health conditions, there are many other small achievements associated with attending a group such as the choir, including getting out of bed and leaving the house:

*For a number of people, you have to get dressed: an achievement. You have to be clean: an achievement. You have to get there, an achievement, and then you meet people. So you're achieving with a structure that you set out. It would have been so easy for me just to lie in bed. I know when I'm struggling, I just lie in bed and I know that if I've stayed in bed for more than a day, I've got a big problem. So it's that bit about that structure of having do something, to get somewhere.* (Wendy; INT)

Sessions at the gardens also provided a sense of achievement and satisfaction, due to the visible impact of the work, for example harvesting vegetable patches or building a trellis. Terry described this to me, especially in the context of his physical disability which means he lives with chronic pain. He had originally felt this disability would severely limit his activities, but found he could do more in the gardens than he thought he would be able to, and this provided him immense satisfaction:

*Terry: If they give me something to do and there*’ *s a possibility it can be finished in that session, then I’ll do my damnest to finish it. I feel disappointed with myself if I leave and it*’ *s not finished yet!*

***Experience of pleasure.*** One of the key shared mechanisms was simply the pleasure and joy members experience during sessions. As Harriet (who suffers from anxiety) from the choir told me “ *It*’ *s just a joyous time, really”.* The group sessions seemed to help reduce individuals’ stress levels. James, a long-standing member of the choir with a history of suicide attempts and recurrent depression, still felt this effect after the sessions:

*It's 'cause I feel good about what we do in the choir and sing, and I go away with this, I can't describe the feeling, but it's like everything lifts inside you, even now. You can go into singing and be stressed out from work or whatever, and by the end of it you're uplifted.* (James; INT)

***Building a new identity.*** Some members talked about how the groups helped them build a new and different identity. For example, before joining the reading group, Meera (who experiences chronic depression and a difficult home life) described her identity as very much centred around her role as wife, mother and grandmother, and providing for others. Over the past several years, she had rarely done things for herself and the group afforded her the opportunity to build a new identity as a reader, who could engage in intellectual discussions with interesting people:

*I would like to be sophisticated, and having joined with the group I'm looking at building my personality, which would have happened but didn't* ‘ *cause of what happened age 21* [entering an unhappy arranged marriage], *and I'm now 60 plus, but I'm still willing to be the Meera that I want to create, I would like to create, and I would say, this team of people, this group has got some great people, and I'm really I'm very grateful that I can associate and communicate because at home there's no communication where I could share.* (Meera; INT)

Meera often talked to the group about the new person she was trying to be, and how the group was helping her build certain aspects of her personality that she had neglected over many years of putting other people’s needs over her own. Yasmeen, the lead facilitator of the group, also described Phillipa’s journey (who had anxiety related to severe chronic pain) and how she felt the book club had the potential to shape a new identity for her:

*It*’ *s that sense of identity. Take someone like Phillipa, who*’ *s had to be a professional patient and had to battle through being ignored and ferocious pain. But when she comes in, she*’ *s like a diva, and she*’ *s got a great gift with poetry, and I*’ *m really hopeful that it allows her a separate identity, separate to that one, and also the one taking care of family members and all those other things.* (Yasmeen; INT)

***Increased purpose/meaning.*** Another mechanism linked to psychological growth, was how the groups provided a renewed purpose and meaning. Sammy (who has paranoid schizophrenia) and Eddy (who has paranoid schizophrenia and history of substance misuse) had each spent a significant amount of time in a mental health hospital prior to being referred into the football group. The club became a way to re-build purpose into their lives after re-entering society:

*The football itself has definitely helped in so many ways because it*’ *s given me a sense of purpose, you know, being around people with the same sort of issues, the same sort of goals* (Sammy; INT)
*It gives you so much more than just football, it gives you a purpose, it*’ *s a way of life.* (Eddy; INT)

This was particularly important for many members who were not working, and the groups provided a purpose often associated with meaningful employment. Moreover, having a purpose provided a reason to get out of bed and out the house, crucial for individuals experiencing mental health difficulties, as Anna (who has PTSD and depression) from the gardening group told me:

*It gives me a sense of purpose. Before, I had no reason to get up.* (Anna; INF)

### Social

***Increased social support.*** Peer support was an important element of the groups, both emotional and practical. Tom (who has a recurrent history of depression and loneliness) and Simon (who has cerebral palsy and associated psychosocial issues) had grown very close through the reading group, and Tom always walked with Simon to the bus stop after group sessions, to support with his physical disability which severely impairs his walking. Moreover, Simon spent several months in hospital during my time with the group, due to respiratory issues related to his disability, and Tom would call Simon each week (physically visiting was not possible due to the Covid-19 pandemic). Providing support for others in the group is something that members found very rewarding. Tom would often talk to people after sessions, or phone or email them, if he observed them being withdrawn or anxious:

“ *I can tell just by listening to someone*’ *s tone of voice, whether they are happy or not, and if I feel it*’ *s the right thing to do then I’ll quite possibly contact them after the group”.* (Tom; INT)

Some of the peer support that occurred was related to the lived experience of mental health problems that volunteers had, or through sharing more practical advice, as Stephanie (director of the gardens) told me:

*The peer support between the different volunteers is quite phenomenal. You know, I’ve never claimed unemployment, I’ve never had to go to a PIP assessment… so there’s a whole sort of level of support which volunteers give to each other because they’ve actually got lived experience.* (Stephanie; INT)

In the football group, I witnessed evidence of peer support in nearly every session, for example players ‘checking in’ with one another, or comparing experiences of their conditions or mental health treatment:

*It*’ *s not like coming to a psychologist and saying I*’ *m having these problems. It*’ *s more like coming to friends and saying I*’ *m having these problems, what experiences have you had with them, or what kind of experiences have you had?* (Sammy; INT)

***Formation of friendships & reduced loneliness.*** For Simon, the people in the reading group and the friendships that formed were the most important aspect of the group:

*It's the people that want me to come back. If hypothetically Yasmeen were to stop doing the group, if Jane stopped doing the group and if everybody were to leave then to me next week, it would be a totally different group, it wouldn’t be the same. You know it is the fact that we have got so close as we have, that we do get on so well, what makes it so successful. And quite honestly, we could be reading this book or we could be reading Noddy. And quite honestly, it wouldn't matter.* (Simon; INT)

The opportunity to form friendships was profoundly important for many members who had often experienced extreme social isolation. For Terry (who has a history of severe alcoholism and experiences loneliness), the gardening group was often the single social interaction he had each week (he attended 2 sessions per week) “ *I don*’ *t really see anyone outside the farm*”, “ *the only thing I do all week is come to the farm*”:

*It started off as just volunteering. And then it became, you know, you get to know people, you get on with people, and you make friends from it… I consider everyone that goes there my friend* (Terry; INT)

Often these evolved into friendships outside the groups too (perhaps a good indicator of genuine friendship), as Loraine from the gardens told me:

*I do enjoy the friendships that go down there. And I have met up with people outside the farm as well… and outside, it feels the same.* (Loraine; INT)

The social side of the football club was of central importance to the mental health benefits of players, and in motivating players to continue attending. For many people, this was about forming lasting friendships.

*I think making good friends that last long is something that*’ *s quite difficult to do. But the club kind of facilitates that, by giving us a common ground and makes us all sort of equals with mental health problems. And so it means you can make lasting friendships.* (Sammy; INT)

***Enhanced sense of community.*** Participants often talked about the sense of community they felt from regularly attending the groups. In the gardens, this was something that some volunteers had never felt much before, despite living locally, as Anna explained to me:

*It did instil in me a bigger sense of community. I love that people get together and do things. I didn’t know there was a farm here, and it*’ *s just down the road to me! I’ve lived here for many, many years and I didn’t know.* (Anna; INT)

Jason, who joined the gardens after his brother died (his brother, who he lived with, had been a very active volunteer there for many years), talked to me at length about how difficult he had found it to make local connections, in such a busy city. For him, the gardens became a vessel in which to do that, allowing him to be “ *much more connected to the local community”,* making connections he “ *wouldn’t have had before”.* Jason’s brother had been his primary social connection in the area and when he died Jason became extremely socially isolated, so this new community was very important for him. He also felt this was a way of continuing his brother’s legacy and helped his grieving process.

The choir provided a sense of community for members, many of whom may not have friends or family living locally. It allowed individuals to feel part of something greater than themselves and a sense of belonging. Often this is possible through work, but many members were unemployed, either due to psychosocial reasons or being retired.

*I didn't do any activities where there was a sense of community until the choir fulfilled that sense of community.* (Christina; INT)

### Behavioural

***Reduction in addictive behaviours, building healthier habits.*** Because of their involvement with the groups, individuals often experienced changes in physical health and health-related habits. For example, Steve in the football group, who was suffering with PTSD and ongoing struggles with drug and alcohol addiction, told me football motivated him to reduce substance use:

*Going (to) football was so powerful. You*’ *re preparing yourself the night before, get your kitbag out, eat some proper food, go to bed right. No drink, I’ve got football. Before I had to get out this house all the time, but once you do football, you*’ *re actually happy and content to recover (here in the house). To sit, not think about drugs, not think about alcohol. You*’ *re actually calm.* (Steve; INT)

Terry from the gardening group, recently recovering from chronic alcoholism, was reluctant at first but eventually came to a session, and after just a few weeks, began to love it, describing it as “ *the best thing I’ve ever done for myself”*, and believing “ *it helped me stay off the drink more than anything else”.* He found this was related to the social element, being around kind people (who are not “ *drinking friends”* and so a more positive, healthy influence on him), being in nature, and the purpose that came from volunteering.

Stephanie, director of the gardens, felt strongly that she is keeping people out of GPs’ and A&E waiting rooms (by reducing harmful, addictive behaviours, and from the physical health benefits of gardening) from her years of experience with the volunteers, but told me this was difficult to “ *prove”*, especially as a small charity with limited capacity:

*We can*’ *t put a value on it, it*’ *s not possible. That*’ *s one of the frustrating things as a small charity. I mean, I’ve been doing presentations to a group of GPs, and I’ve said, hand on heart, I know that our work with volunteers is keeping people outside your waiting room and one of the GPs put their hand up and said, well how can you prove that? And I know it from talking with the volunteers but I can never actually, you know, demonstrate by facts and figures because we don*’ *t have the capacity to do that kind of research. And I don*’ *t think it*’ *s appropriate, I think it would put people off.* (Stephanie; INT)

***Increased work-seeking behaviour.*** Participants across all groups spoke about how their involvement with the groups helped their motivation to seek work and employability, for example Sammy from the football group:

*It*’ *s very beneficial for me, I*’ *m re-using skills that I’ve already learned, as well as being something I*’ *m able to put on my CV.* (Sammy; INF)

Mahmoud from the football group has a diagnosis of paranoid schizophrenia related to excessive drug use, spent 7 years in the local mental health hospital and time in prison related to drug offences, and told me the club was “ *magical”*, having helped him realise he would like to work, preferably in an area that “ *gives back”* to society.

*Right now they*’ *re helping me with a DBS reference,* ‘ *cause in the future I want to work.* (Mahmoud; INT)

Several volunteers also found the gardens helped them re-enter the world of work or make plans to re-enter. Many of the volunteers were unemployed, often due to mental health or social reasons. For example, Anna has been unemployed for several years, and previously unable or fearful of re-entering the job market due to her PTSD, but found she was more motivated because the gardens acted as that “ *stepping-stone”* to building her self-confidence:

*I applied for this job I’ve been interested in for 3 years. I didn’t get an interview, but it was still huge, a big deal. I just took the bull by the horns. I spent 5 days researching and getting ready for it.* (Anna; INT)

***Provision of structure, routine.*** Another important theme in supporting the well-being of individuals was the structure or routine that regular engagement with their group provided. Most members who attended regularly were not in work, often due to mental health reasons, thus the importance of finding positive activities to form a routine was tantamount to their well-being. Dave from the football group, who struggles with recurrent depression, described this:

*If you*’ *re suffering with depression, sitting with yourself for long periods without anything in terms of a purpose in mind will generally lead you to (further) depression because your thinking being prone to negativity will take you down that path. Now, if you have sort of things set out in front of you in terms of like a stable routine, it can be a very strong adversary for depression. Structure is very important.* (Dave; INT)

Further, he talked about how just getting out the house, which can seem near-on impossible when in certain mental health states, can be made slightly easier when having a structured, familiar activity to look forward to:

*Going out the front door can be a nightmare, you know? But overcoming that obstacle with something that*’ *s familiar makes it easier, you know? It*’ *s like, okay, I*’ *m going there, I know people that are there, I know what*’ *s going on there, it*’ *s going to be fine, just get out the door.* (Dave; INT)

Mahmood expanded on this, too, articulating the importance of keeping busy, and having something to look forward to:

*It would be really difficult to get out of bed if I didn’t have something like football, because of giant feelings of loneliness and low mood and stuff. So a big part of it is just having some kind of meaning to keep busy and something to look forward to each day.* (Mahmoud; INT)

Sandy, one of the social prescribing link workers that refers into the gardens group, agreed that structure seems to be one of the most important beneficial elements to these groups:

*I think one of the key things when referring people is the kind of structure, I think that*’ *s what people are looking for. Some people want to fill their week with like a timetable of activities. If you’ve got to wake up because you*’ *re doing that activity on this day, I think, yeah it definitely provides that routine and structure.* (Sandy; INT)

***Increased independence & openness to experience.*** The groups often inspired members to try new hobbies, activities and generally live life more fully. In the reading group, this seemed to be a combination of inspiration from the literature (especially the poetry) as well as from other group members and facilitators. India (who has anxiety and depression and chronic, low self-confidence) had started volunteering:

*I was applying to do, um, voluntary work at a community centre. Yeah, which I never would have thought of doing before. Um, so I think the group's give me a bit of confidence to, you know, go forward and do that.* (India; INT)

Meera from the reading group decided to plan a holiday for herself, because of the encouragement of the group, something she’d never felt able to do before. India also found that the group had given her “ *the encouragement to explore different things”.* For Kylie (who experiences loneliness, depression and PTSD) the choir had a significant impact on her motivation and ability to engage more in society in general, and live life more fully.

*(My life) has changed a lot. Yeah, because of the past problem, I didn't want to get close to anyone, if it wasn't for the choir I wouldn't be able to go out the house. Now, I would like to sit in the bus, and go somewhere… before I wouldn't have talked to anyone, now I talk to people.*
*I always relied on people. Now I have realised I can do things for myself.* (Kylie; INT)

## Discussion

This study has illuminated the key individual-level mechanisms that lead to improved psychosocial well-being across four social prescribing community groups for people experiencing mental health problems. Our findings support the conceptualisation of social prescribing activities as complex interventions ( Craig *et al.*, 2008), involving many different mechanisms which produce mental health benefits. Whilst it was not possible to discuss in-depth every single mechanism identified (See *Extended Data* ( Aughterson, 2024) for a full list of mechanisms), there were a number of shared mechanisms between the groups. Although it may not be possible to draw any definitive conclusions as to the wider universality of such shared mechanisms, given that the four groups were purposively chosen to represent a wide breadth of social prescribing categories, we argue there is some utility in illuminating the more prescient themes.

As outlined above, the observed activities provided enjoyment and pleasure for participants. Often it was helpful if a participant had enjoyed the activity before, indicative of previous social prescribing research findings ( Husk *et al.*, 2020), however this was not necessary in all cases. The pleasure component is likely shared across most social prescribing community groups more widely, and arguably sets these groups apart from most professional therapeutic support.

All of the groups provided a positive outlet, where participants could develop various skills related to the specific activity and more broadly, the ability to socialise in a ‘safe space’, relatively free of judgement, which in turn led to increased self-confidence and self-esteem. Improved confidence and self-esteem in themselves are arguably valuable endpoints but are also mechanisms directly correlated with improved mental health outcomes ( Moksnes & Reidunsdatter, 2019; Owens, 1993). These groups seemed to act as a ‘stepping stone’ for individuals to improve their life more broadly. Much of this is first centred around improved self-esteem and self-confidence, from which individuals develop better social skills, motivation to seek work, build healthier habits, increased openness to new experiences and greater independence – all of which are mechanisms that can contribute to greater psychosocial well-being ( Fancourt *et al.*, 2021). These mechanisms suggest that individuals become more ‘activated’ in their own health and well-being, an idea that the recent concept of ‘patient activation’ and earlier theory of ‘self-efficacy’ explore ( Bandura, 1978; Hibbard & Greene, 2013). While both of these concepts have associations with long-term improvements in mental health outcomes, the evidence on patient activation’s relationship with mental health is still in the preliminary stages, with research so far focused on physical health outcomes ( Hibbard & Greene, 2013; Schönfeld *et al.*, 2016).

Individuals often described the purpose and meaning the groups brought to their lives. Seligman, founder of the Positive Psychology movement, defines meaning or purpose as ‘belonging to, or serving, something greater than ourselves’ ( Seligman, 2012). Purpose and meaning in one’s life are now considered fundamental to flourishing mental well-being, rooted in increasingly Eudaimonic thinking on mental health and reflecting a departure from purely hedonistic conceptualisations of happiness ( Deci & Ryan, 2008). Some of the most successful theories of well-being now incorporate purpose or meaning, e.g. Ryff’s Psychological Well-Being (PWB) ( Ryff & Keyes, 1995) and Seligman’s PERMA theories of well-being ( Seligman, 2018). Relevant to individuals in our study, many of whom have had substance misuse problems, there is evidence that ‘having a purpose’ can be a crucial component in helping tackle addiction ( Lyons *et al.*, 2010).

The social component to these groups is also fundamental. Members experienced lasting friendships, more positive influences, an enhanced sense of community, increased social support, and reductions in loneliness. The importance of forming friendships, especially for those who are chronically lonely, is fundamental, and arguably should be considered a worthy outcome in itself. There is also substantial evidence for the impact of social connection on mental health, physical health and mortality ( Age UK, 2018; Cacioppo *et al.*, 2002; Hawkley & Cacioppo, 2010). Our findings corroborate previous research on social prescribing’s potential to facilitate friendship formation and reduce loneliness ( Haslam *et al.*, 2016; Kellezi *et al.*, 2019; Kimberlee, 2016).

The types of social connection are important too, as Putnam’s work on negative social capital (e.g. smoking or drinking networks) vs positive social capital highlights ( Putnam, 2000; Rohman, 2014). These groups seem to fall more within the positive social capital category, containing individuals who are mostly positive, healthy influences on each other’s life. This is especially important among those with addiction problems. Besides addiction, for those with mental health difficulties more broadly, being surrounded by positive influences (mostly “kind people” with similar difficulties and shared goals) helped foster supportive habits and attitudes towards one’s mental health, as seen in the building healthier habits and increased openness themes.

An arguably novel, supplementary finding to this research is the evident utility of these groups for individuals with severe mental illnesses. Some members with the most severe problems were still accessing therapy and/or medication alongside their group, but the groups themselves still seemed to be responsible for profound benefits. Previously, most social prescribing studies have focused on those with mild to moderate mental health conditions ( Bertotti *et al.*, 2017; Bickerdike *et al.*, 2017; Carnes *et al.*, 2017; Dayson & Bennett, 2016; Friedli *et al.*, 2012; Kimberlee, 2013), and it has been suggested that these groups may be less suitable for those with more severe problems ( Drinkwater *et al.*, 2019). This is also reflected in national policy, with NHS social prescribing being largely rolled out throughout General Practice, and link workers employed via GP primary care networks ( Taylor & NHS, 2020). The Royal College of Psychiatrists, however, has recently argued for the utility of social prescribing for mental health service users and in-patient settings, and called for more training for psychiatrists in social prescribing ( RCPSYCH, 2021). Our study supports this call, showing that it is important for those with diagnoses of severe mental illness to be given the opportunity to access and benefit from such groups, where possible.

## Limitations

There were several limitations to this study. Firstly, there was potential for group selection bias – all four groups were very keen to partake in the research, had a strong online, social media presence, and were long-standing, relatively well-known groups. They may therefore have been more likely to be ‘successful’, or better resourced community groups compared to others (all the groups were also based in London, which may further limit the generalisability of the findings). Additionally, considering the focus was on the potential psychosocial *benefits* of these groups, this research will have missed some key negatives and challenges that groups might face and it is important future research explores these.

Further, there was possible self-selection bias in terms of who the ‘long-term research companions’ and interviewees were, who form a significant proportion of the findings. These individuals did reflect a wide range of mental health conditions and psychosocial difficulties; however, it is possible that those who more actively participated in the research were different to those who did not, e.g. they may have had more positive experiences of the groups.

Moreover, due to the Covid-19 pandemic, the singing group in particular was significantly impacted. This meant that a significant proportion of the research was via virtual format. Therefore, emergent properties of the singing group may have been missed that would occur in-person, e.g. certain types of socialising and social support. Fortunately, these could be explored in-person for the last 4 months of the study. The use of in-depth interviews was also especially important in this group for this reason, as was regular attendance at the choir’s in-person, weekly walking group.

Lastly, biological mechanisms were not explored; a crucial component of the leisure mechanisms framework. Future research should explore these mechanisms using biomedical measurements. Some biological mechanisms that were observed throughout the ethnography in one or two groups are however included in the *Extended Data* ( Aughterson, 2024), e.g. weight loss, reduced pain, and improved physical health symptoms. Moreover, this study focused on micro-level mechanisms, and there will be many meso- and macro- level mechanisms at play too that could be an area for future research. Further, a combination of active ingredients (e.g. facilitator inter-personal skills or physical activity) and moderators (e.g. gender, ethnicity, geography, etc) are responsible for which mechanisms are activated in individuals. Thus, future research should also explore key active ingredients across different social prescribing community groups, and the degree to which various moderating factors influence engagement.

## Conclusion

This study illuminated shared individual-level mechanisms leading to improved psychosocial well-being across four social prescribing community groups. Important commonly shared psychological mechanisms were: increased purpose/meaning, experience of pleasure, increased self-confidence & self-esteem, building a new identity, and increased sense of achievement. Social mechanisms were: increased social support, formation of friendships and reduced loneliness, and enhanced sense of community and belonging. Behavioural mechanisms included: provision of structure/routine, reduction in addictive/unhealthy behaviours and building healthier habits, and increased independence and openness to experience. Such mechanisms should be considered by GPs and other referring professionals when considering potential benefits to patients. These mechanisms also provide valuable targets for future research into the impact of social prescribing schemes and how social prescribing interventions impact on health outcomes. This study also highlighted the importance of these groups for supporting people with more severe mental illnesses, which future policy, funding and training for referrers should reflect.

## Ethics and consent

Following transcription, original recordings of interviews were deleted, all participants and names of people mentioned in the transcripts were given pseudonyms to preserve their anonymity, and all specific locations and identifying features removed. The study was reviewed and approved by the Camden & Kings Cross Research Ethics Committee (279076; 20/LO/1214; 14.12.2020). Capacity to consent to be involved in the study applied the principles of the four-step Mental Capacity Act ( SCIE, 2017).

## Data Availability

Further data including a full list of mechanisms, interview guide, and further background to the groups can be accessed as *Extended data*. For some data, e.g. transcript data, it was not possible to anonymise fully in order to protect the security of participants and groups and so this has not been uploaded to the online data repository, however certain anonymised transcript data can be made available on reasonable request. Open Science Framework: Social prescribing ethnography. https://doi.org/10.17605/OSF.IO/73JWX ( Aughterson, 2024). This project contains the following extended data: Appendix A (Extended list of mechanisms across the four groups) Appendix B (Interview guide) Appendix C (Background to the groups) Data are available under the terms of the Creative Commons Zero "No rights reserved" data waiver (CC0 1.0 Public domain dedication).
