# Supplementary material for: Social prescribing for individuals with mental health problems: An ethnographic study exploring the mechanisms of action through which community groups support psychosocial well-being
Source: Wellcome Open Res. 2024 Mar 19;9:149. [Version 1] doi: 10.12688/wellcomeopenres.20981.1 (PMC11216282; doi:10.12688/wellcomeopenres.20981.1)
Supplement: Peer review history

# Reviewer report: Reviewer response for version 1

**DOI:** 10.21956/wellcomeopenres.23214.r82205

**Published:** 2024-06-28

**Authors:** Genevieve Dingle

**Affiliations:** 1 School of Psychology, The University of Queensland, Saint Lucia, Queensland, Australia

Thank you for the opportunity to review this paper which describes an ethnographic study of 4 community group programs offered as part of social prescribing, to examine the mechanisms by which such groups might impact on participants' mental health outcomes. The first author attended the four groups weekly, observing, taking notes, and conducting interviews over a 12-month period. I applaud the author for making such a substantial investment of time and for generating rich data about the experience of participants in these groups.

 The introduction describes social prescribing and identifies the gap in knowledge that will be addressed by the study that is, understanding the mechanisms by which engaging in community group programs through social prescribing may produce effects on participants' psychosocial well-being. There have been previous studies of the mechanisms by which community groups effect psychosocial well-being of adults (e.g., from my own group, see Dingle et al., 2013 http://pom.sagepub.com/content/41/4/405; Williams et al., 2020 https://doi.org/10.1080/17533015.2019.1624584; Draper et al., 2021, https://www.frontiersin.org/journals/psychology/articles/10.3389/fpsyg.2021.646292/full). A potential new aspect here would be understanding what a referral from a health provider and a relationship with a community connector / link worker might add to the mechanisms within the group program itself, but these relational aspects of social prescribing do not seem to be part of the current study?

 I take the author's point that a comparison of experiences by type of mental illness is not warranted here, but I'm unclear about the rationale for analysing 'key shared mechanisms' across the four types of groups'  i.e., football, choir, reading group, gardening group. Maybe some mechanisms are more or less salient in the various types of groups or not found in some groups, and this would be interesting to know. I also wonder if the experience of participants versus facilitators or service managers may have been quite different?

 The data were analysed using reflexive thematic analysis, although I didn't see a reflexive statement from the first author which would help to understand the 'lens' through which they perceived and organised the data. The inductively generated codes were classified into psychological, social, and behavioural mechanisms during the analysis and write up phases, aligning with the multi-level theoretical framework of mechanisms of action for leisure activities (Fancourt et al., 2021).

 The results include lots of great quotes from participants to illustrate the various mechanisms at play. (Despite the different methodological approach and location of this study, there is remarkable overlap with the themes and subthemes in the aforementioned papers.) Nice links here with other theoretical frameworks such as the PERMA model from positive psychology and the psychological needs satisfaction from Deci & Ryan (see also Draper et al., 2021 https://www.frontiersin.org/journals/psychology/articles/10.3389/fpsyg.2021.646292/full and a review of theories used in social prescribing research by Evers et al., 2024 https://bmchealthservres.biomedcentral.com/articles/10.1186/s12913-024-10563-6).

 The behavioural mechanisms are relatively new in this context and more could be said about this category. There are links here with previous research about the role of social groups in addiction recovery ' e.g., Best et al., 2015, https://doi.org/10.3109/16066359.2015.1075980

 Minor point,  the term 'openness to experience' is a well-established personality factor from the 'Big Five' (e.g., https://journals.sagepub.com/doi/abs/10.2190/H8H6-QYKR-KEU8-GAQ0), and I don't think that's quite what the authors are referring to here. Perhaps give that subtheme a different label?

 Overall, an interesting paper that makes a valuable contribution to the research about how non-clinical programs can support people's psychosocial well-being.

Is the work clearly and accurately presented and does it cite the current literature?

Partly

If applicable, is the statistical analysis and its interpretation appropriate?

Not applicable

Are all the source data underlying the results available to ensure full reproducibility?

Yes

Is the study design appropriate and is the work technically sound?

Yes

Are the conclusions drawn adequately supported by the results?

Yes

Are sufficient details of methods and analysis provided to allow replication by others?

Yes

Reviewer Expertise:

Clinical psychology; music psychology; social prescribing; mental health

I confirm that I have read this submission and believe that I have an appropriate level of expertise to confirm that it is of an acceptable scientific standard, however I have significant reservations, as outlined above.

# Reviewer report: Reviewer response for version 1

**DOI:** 10.21956/wellcomeopenres.23214.r82203

**Published:** 2024-06-18

**Authors:** Helen Quirk

**Affiliations:** 1 The University of Sheffield, Sheffield, England, UK

This is a qualitative ethnographic study exploring the individual-level mechanisms underlying how social prescribing to community groups support psychosocial wellbeing among people living with severe mental illness. It looked for shared psychosocial mechanisms across 4 leisure activities – football, singing, gardening and reading.

**Overall feedback**

 This is a well written manuscript describing an important and interesting topic. The ethnographic researcher should be praised for overcoming the challenges of the Covid-19 pandemic, which must have caused some problems for the programme of research and ethnographic approach.

**Abstract**

 Some detail was lacking in the Abstract. The Methods states an ethnographic study but lacks detail about what was done, where and how many participants were involved.

 The methods also mentions a ‘multi-level theoretical framework of mechanisms of action’, putting this in inverted commas (though it’s not clear why) but offers no explanation about what this is or how it framed the findings.

**Introduction**

 Paragraph 2 states that “fewer studies focusing on individuals with severe mental illness”. It would be good for the authors to explain why this is alongside a justification for the focus on severe mental illness in this study.

 Paragraph 4 describes the ‘multi-level leisure mechanisms framework’. Firstly, does this framework need a reference/citation other than Fancourt et al 2021 or was it developed by Fancourt et al? Secondly, the authors describe the development of the framework from a literature review. More detail could be provided about this review – it isn’t immediately clear that the authors are referring to the Fancourt et al 2021 paper again. It would be good to have a bit more detail about the population upon which this framework was developed and how applicable that is to severe mental illness.

 The authors state that their focus in this study was on the individual (micro) level. A rationale/justification for this would strengthen the argument and would help support the overall rationale for this study.  

 The final paragraph of the introduction states “Therefore, this study explored mechanisms of action across four different social prescribing community groups for adults with mental health problems”. Please specify here that you mean severe mental illness, if this is correct.  

**Methods**

 The Methods section provides a nice explanation of the study design and offers sound rationale for the setting (groups chosen), although a justification for not focusing on groups focusing on financial/welfare support would be beneficial.  More detail around who the groups were targeted at would also aid the readers interpretation of findings – for example where the groups open to the general public or specifically for people with mental health conditions? Were they groups delivered by or via the NHS or were they independent of health services?

 Clarity around the population / participants is needed. It is explained that group members had a diagnosed mental health condition and / or significant psychosocial challenges. Earlier in the manuscript it was stated that the focus of this study is on severe mental illness- clarity and consistency is needed in the description of the sample and the authors should provide a definition of what they mean by severe mental illness.  

 In the Participant characteristics table in Table 2- Rather than stating ‘ethnic minority’, it would be more appropriate to state the ethnic groups those 9 participants belong to, if that information is available.

 Description of the Methods implies that the focus of this ethnographic study was on the community groups themselves, rather than on the social prescription to these groups. The manuscript might benefit from being reframed to reflect this, or the authors might wish to provide a stronger rationale / explanation for how social prescribing is being studied.

 The authors provide a strong rationale for using Reflexive Thematic Analysis. Given the importance of positionality, it would be beneficial to provide details of the authors (background, experience and relationship to participants) to give more transparency. Reporting may benefit from referring to the CORE-Q checklist for reporting qualitative research.

**Results**

 The multi-level leisure mechanisms framework provides a nice structure around which to present the findings, but I do wonder whether this is at odds with (goes against the premise of) Reflexive Thematic Analysis, where Braun and Clarke caution against using a rigid framework or predefined topics. My concern is that some of the richness and complexity from the inductive, reflexive analysis might be lost in the presentation of the findings within the framework. For example, did any of the coding and emergent themes fall outside of this framework and is there scope to present these themes in the manuscript?

 Related to this, I have a concern that the theme names describe general or broad topics covered in the data rather than underlying issues and patterns within the topic. For example, the theme title “Building a new identity” could provide more description from the data e.g., “Groups instill confidence among attendees to build a new identity”. This is just a suggestion. I would recommend that the authors consider the theme names (and perhaps themes themselves) to ensure that they capture the essence of the data rather than just categorising it.

 Taking the themes ‘Increased self-confidence & self-esteem’ and ‘increased sense of achievement’ – the authors present a strong case for the presence of these psychological mechanisms as contributing mechanisms of action, but I think the authors could go deeper by explaining how the community groups/activities increased them. What was it about the specific qualities of the group that brought about those mechanisms of change?

 Minor point, please define “PIP assessment” in Stephanie’s quote under “Increased social support”.

**Discussion**

 Overall the Discussion is good and makes some important points and inferences from the data.

 In the paragraph about severe mental illness specifically, it would be worthwhile referring to some literature that describes the experience of community (support) groups for people with severe mental illness to draw out any similarities or differences with the findings in this study.

 I would like to see some discussion around the utility of the multi-level leisure mechanisms framework for this population group.

**Limitations**

 The findings are overwhelmingly positive, when we know that the journey to actually getting to these groups (physically and mentally) can be turbulent, long and full of set-backs for people living with severe mental illnesses. I am pleased that the authors raised the possible limitations of this ‘positive lens’ (i.e., focus on benefits) as a limitation of the study.

 The final limitation recognizes that some of complexity of the mechanisms of change (e.g., combinations of active ingredients – and interaction with different contexts) might be lacking in this study. Future research could consider using a realist approach which would seek to understand what works, for whom, under what circumstances and why. Such an approach would allow this complexity to be explored with consideration to the context in which they happen.

Is the work clearly and accurately presented and does it cite the current literature?

Partly

If applicable, is the statistical analysis and its interpretation appropriate?

Not applicable

Are all the source data underlying the results available to ensure full reproducibility?

Yes

Is the study design appropriate and is the work technically sound?

Partly

Are the conclusions drawn adequately supported by the results?

Yes

Are sufficient details of methods and analysis provided to allow replication by others?

Yes

Reviewer Expertise:

My area of expertise is physical activity for health. I am a qualitative researcher with expertise in the experience of physical activity for people living with severe mental illness. I am particularly interested in community-based physical activity.

I confirm that I have read this submission and believe that I have an appropriate level of expertise to confirm that it is of an acceptable scientific standard, however I have significant reservations, as outlined above.

# Reviewer report: Reviewer response for version 1

**DOI:** 10.21956/wellcomeopenres.23214.r78787

**Published:** 2024-05-30

**Authors:** Marcello Bertotti

**Affiliations:** 1 Institute for Connected Communities, University of East London, London, England, UK

Summary of the article

 This is an ethnographic study that explored how community groups support psychosocial well-being. It focuses on 4 social prescribing community groups including football, singing, gardening and reading which supported people with severe mental illness. A range of positive psycho-social and behavioral mechanisms were identified which lead to psychological well-being as the main outcome.

 Yes overall. however, I would suggest the following point: It requires a stronger rationale as to how this is actually social prescribing rather than an ethnographic study about the potentially psychological benefits of community groups. There is an initial description of this in the first paragraph of the introduction but the question is not adequately addressed as many of the alternative referral mechanisms cited make us of a link worker. It is important to recognize the diversity of opinions of what constitutes social prescribing. For many people, it involves a ‘link worker’ which is absent from this analysis. The Delphi study on the definition of social prescribing by Muhl et al (2023) may help with this task.

 The study design is accurately presented and unique as it combines 600 hours of ethnographic observations with qualitative interviews with a range of stakeholders, mostly participants to the four community activities. 

 The methods and analysis are well explained and the limitations section is adequately addressed. For instance, the focus on ‘benefits’ of these 4 community groups may have cast a shadow on the more negative processes. For instance, it is well known in the literature that people with severe mental health issues may need a lot of 1-2-1 support before they can join a community group which involves socialisation. This may be a problem specifically for the 6 people experiencing anxiety in this study. This limitation has been recognised. 

 Discussion and conclusions

 The discussion section is overall good and make important points. However, I would suggest the authors to re-considering one point in the latter part of the discussion section. In this section, the authors cited the conclusions from other studies in relation to the unsuitability of social prescribing for those with severe mental health problems. However, such studies examined the social prescribing in its entirety including the role of the link worker in this process. The reason for the focus on mild to moderate mental health issues in these studies is due primarily to the fact that link workers are not seen as adequately trained for referring a person with severe mental health problems to activity groups. This should be qualified in this discussion point.

 Suggestions for future studies would be to include link workers in ethnographic observations as this has only been touched upon in social prescribing to date.

 Another suggestion may be that the mechanisms identified through this study could be used as a basis for further quantitative enquiry over a larger sample of people. E.g. the use of self-esteem scale, social capital harmonised questionnaire set potentially to investigate social support, loneliness scale (de Jong) etc.

Is the work clearly and accurately presented and does it cite the current literature?

Partly

If applicable, is the statistical analysis and its interpretation appropriate?

Not applicable

Are all the source data underlying the results available to ensure full reproducibility?

Yes

Is the study design appropriate and is the work technically sound?

Yes

Are the conclusions drawn adequately supported by the results?

Partly

Are sufficient details of methods and analysis provided to allow replication by others?

Yes

Reviewer Expertise:

My main area of research is community health. I have been involved in evaluations, research, training in the area of social prescribing for the last 10 years.

I confirm that I have read this submission and believe that I have an appropriate level of expertise to confirm that it is of an acceptable scientific standard.
